# Data for characterization of SALK_084889, a T-DNA insertion line of *Arabidopsis thaliana*

**DOI:** 10.1016/j.dib.2017.05.047

**Published:** 2017-05-31

**Authors:** Mingqi Zhou, Anna-Lisa Paul, Robert J. Ferl

**Affiliations:** a Department of Horticultural Sciences, Program in Plant Molecular and Cellular Biology, University of Florida, Gainesville, FL 32611, United States; b Interdisciplinary Center for Biotechnology Research, University of Florida, Gainesville, FL 32610, United States

**Keywords:** Arabidopsis, DREB2A, LOX1, SALK_084889, T-DNA

## Abstract

In this article we report the identification of T-DNA (transfer DNA) insertion sites within two different gene regions in the genome of an Arabidopsis mutant line, SALK_084889. The T-DNA positions are in the 3′ UTR (untranslated region) of DREB2A (Dehydration-responsive element-binding protein 2A) (AT5G05410) and promoter of LOX1 (Lipoxygenase 1) (AT1G55020) as determined by DNA-PCR and sanger sequencing. The expression levels of DREB2A and LOX1 were also analyzed using quantitative realtime PCR (qPCR) in SALK_084889 and wild type Arabidopsis (Col, Columbia). Further, the comparison of drought and heat tolerance between Col and SALK_084889 were conducted by stress treatments. The present data indicate that in SALK_084889, the expression of DREB2A is not downregulated under normal growth conditions but can be affected only in roots under drought treatment, while LOX1 is significantly downregulated in both roots and shoots under all tested conditions. These data are original and have not been published elsewhere.

**Specifications Table**

**Table:** 

Subject area	*Biology*
More specific subject area	*Plant biology*
Type of data	*Figures, Tables*
How data was acquired	*DNA-PCR, Quantitative Realtime PCR (qPCR), Sanger sequencing, Stress treatments, Photograph for plant phenotypes*
Data format	*Raw, Analyzed*
Experimental factors	*SALK_084889 and Col Arabidopsis plants*
Experimental features	*DNA-PCR was employed to identify the T-DNA insertion sites in SALK_084889 genome within DREB2A 3*′ *UTR and LOX1 promoter region, respectively. Then SALK_084889 plants were subjected to qPCR to examine the expression levels of DREB2A and LOX1. The drought and heat responses of SALK_084889 were also performed.*
Data source location	*UF, Gainesville, USA*
Data accessibility	*Data is within this article.*

**Value of the data**

• T-DNA insertion lines provide important resource for genetic analysis based on mutagenesis in plant research. SALK lines are the most widely used T-DNA insertion lines for the model plant Arabidopsis. Accurate assessment of insertions is critical for understanding the value of the insertion lines.
• SALK_084889 is annotated as a T-DNA insertion line of *DREB2A*, a key regulator of drought and heat response in Arabidopsis. We characterized *DREB2A* expression in normal and drought conditions in this line, which is relevant for analysis of *DREB2A* mutants in further investigation.
• The *LOX1* gene plays a critical role in multiple bioprocesses associated with lipid peroxidation. Our data identified a T-DNA insertion within promoter of *LOX1* and showed the knock-out expression of *LOX1* in SALK_084889. These are valuable information for mutation analysis of *LOX1*.

## 1 Data

**Fig. 1:**
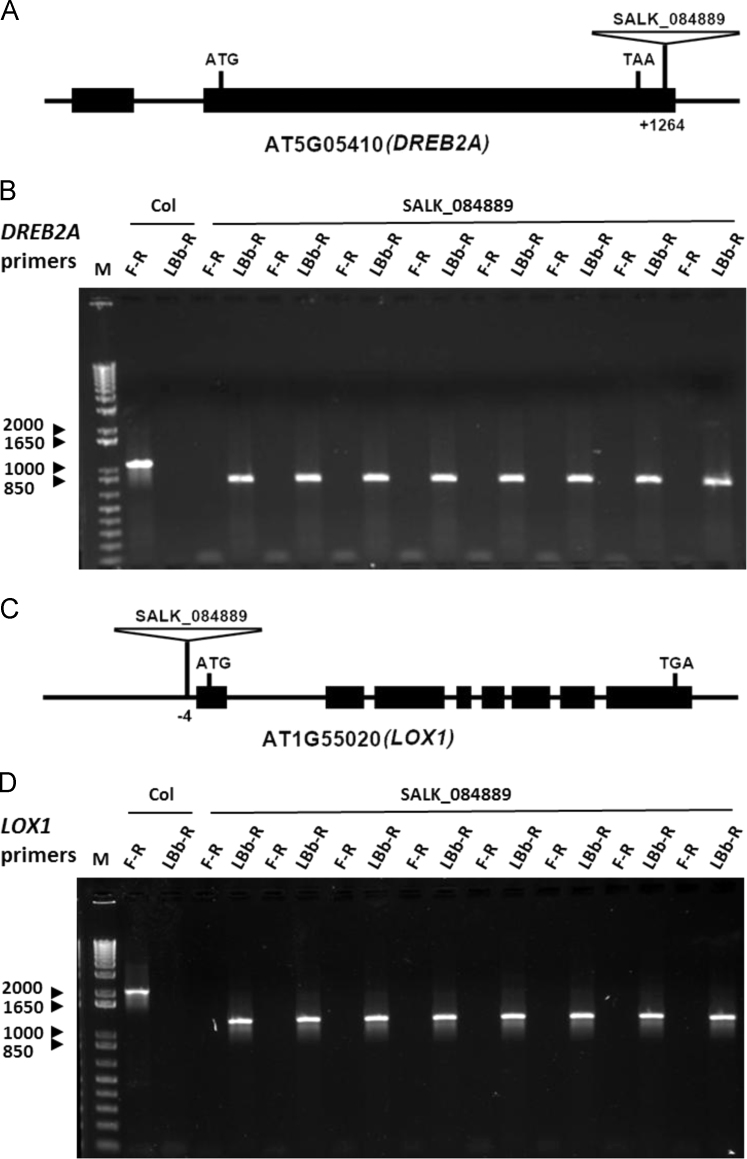
T-DNA insertion in 3′ UTR of *DREB2A* (AT5G05410) and promoter of *LOX1* (AT1G55020) in SALK_084889. (A) Gene structure of *DREB2A*. The T-DNA position is in +1264 bp after transcription start site. (B) PCR amplification of wild type allele band using forward (F) and reverse (R) gene specific primers DREB2A-F and DREB2A-R (F-R) as well as T-DNA band using LBb1.3 and DREB2A-R (LBb-R). One Col wild-type seedling and eight randomly selected SALK_084889 seedlings were used. (C) Gene structure of *LOX1*. The T-DNA position is in −4 bp before transcription start site. (D) DNA-PCR of wild type allele band using gene specific primers LOX1-F and LOX1-R (F-R) as well as T-DNA band using LBb1.3 and LOX1-R (LBb-R). All primers are listed in Table 1. The SEQ files of sanger sequencing are shown in Supplementary material.

**Fig. 2:**
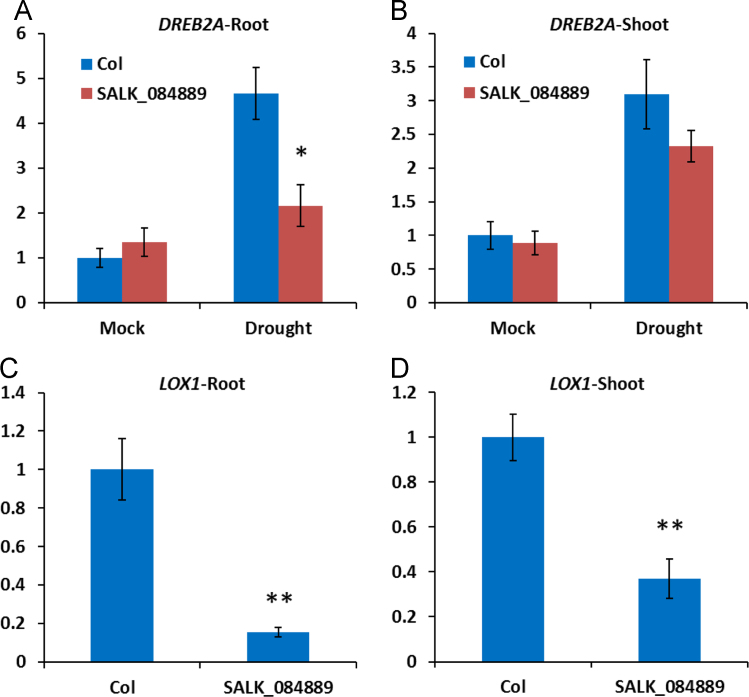
Relative expression levels of *DREB2A* and *LOX1*. The expression of *DREB2A* was determined in roots (A) and shoots (B) of Col or SALK_084889 plants with or without drought treatments. The expression of *LOX1* was determined in roots (C) and shoots (D) of Col or SALK_084889 plants in normal condition. The expression levels of *DREB2A* or *LOX1* in Col in normal conditions were initiated as “1” so that relative expression levels of other samples were determined. The *UBQ11* (AT4G05050) gene was used as the internal control. The Ct (cycle threshold) values are shown in Supplementary table 1. Data are means±SE (*n*=3). The student׳s *t*-test was performed to show the significant difference of gene expression between Col and SALK_084889 (* *p*<0.05, ** *p*<0.01).

**Fig. 3:**
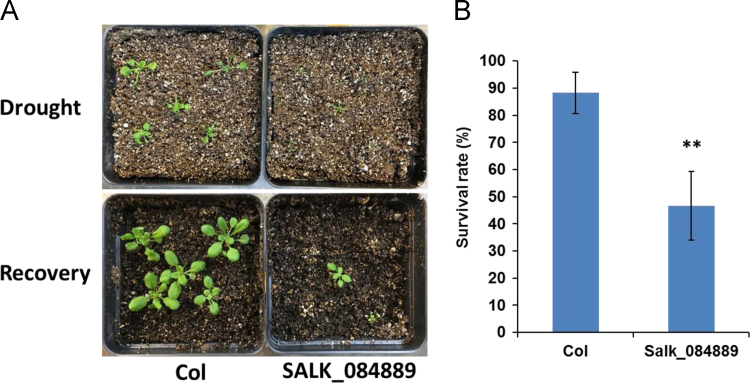
Drought tolerance of SALK_084889. (A) Phenotype of Col or SALK_084889 plants after drought treatment followed by recovery. (B) Survival rate of Col or SALK_084889 plants treated by drought. The number of survived plants are shown in Supplementary table 2. Data are means±SD (*n*=3). The student׳s *t*-test was performed to show the significant difference of survival rate between Col and SALK_084889 (** *p*<0.01).

**Fig. 4:**
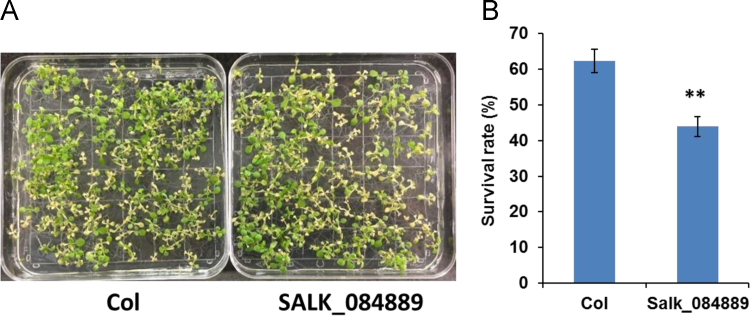
Heat tolerance of SALK_084889. (A) Phenotype of Col or SALK_084889 plants after heat treatment followed by recovery. (B) Survival rate of Col or SALK_084889 plants treated by heat. The number of survived plants are shown in Supplementary table 2. Data are means±SD (*n*=3). The student׳s *t*-test was performed to show the significant difference of survival rate between Col and SALK_084889 (** *p*<0.01).

The dataset of this article provides information on T-DNA insertions in SALK_084889. Fig. 1 shows the T-DNA bands amplified within *DREB2A* (AT5G05410) and *LOX1* (AT1G55020) genes of SALK_084889 as well as T-DNA insertion sites determined by Sanger sequencing. Fig. 2A–B show *DREB2A* expression in both roots and shoots in normal conditions with or without drought treatment. Fig. 2C–D show *LOX1* expression in roots and shoots in normal conditions. Fig. 3, Fig. 4 show the comparison of survival rates between Col and SALK_084889 plants in drought and heat treatments. Table 1 shows the sequences of primers used in experiments for Fig. 1, Fig. 2.

**Table 1:** Primers used for the presented data.

Gene	Name	Sequence (5′-3′)	Usage
DREB2A (AT2G16060)	DREB2A-F	TAAAGGAATGCTTGTTGGTGG	T-DNA PCR
	DREB2A-R	GAGCCGATGTATTGTCTGGAG	
	DREB2A-qPCRF	TGATTTTCAAATTTCGTCCCCTATAG	qPCR
	DREB2A-qPCRR	AGTACCGTCACCTCTACTTCTAG	
LOX1 (AT4G33070)	LOX1-F	CAGAGGAATTTTTAAGCTTTTAAGAC	T-DNA PCR
	LOX1-R	GGAAAAAGTCTTTGAATGTATGCAC	
	LOX1-qPCRF	GGATGGACTCACTGTTGAAGAG	qPCR
	LOX1-qPCRR	TGCATAAGTCTTGGTCGTGG	
N/A	LBb1.3	ATTTTGCCGATTTCGGAAC	T-DNA PCR
UBQ11 (AT4G05050)	UBQ11-F	AGCAACTTGAGGACGGCAGA	qPCR
	UBQ11-R	GTGATGGTCTTTCCGGTCAAA	

## 2 Experimental design, materials and methods

Arabidopsis seeds of wild tyoe (Col) and SALK_084889 were obtained from Arabidopsis Biological Resource Center. SALK_084889 was reported as a T-DNA insertion line of *DREB2A* (AT5G05410) gene [1]. For identification of T-DNA insertion sites, Col and SALK_084889 seeds were grown in soil at 22 °C under constant light condition. Eight randomly selected SALK_084889 seedlings were subjected to DNA-PCR to confirm all seeds are homozygous. For *DREB2A*, the gene specific primers DREB2A-F and DREB2A-R were used to amplify wild type allele band while LBb1.3 and DREB2A-R primers were used to amplify T-DNA band. For *LOX1*, the gene specific primers LOX1-F and LOX1-R were used to amplify wild type allele band while LBb1.3 and LOX1-R primers were used to amplify T-DNA band. The amplicons of T-DNA bands of *DREB2A* and *LOX1* were purified by subjected to sanger sequencing and the alignment of sequences was performed using Vector NTI v10.0 program (InforMax Inc). For Expression analysis, Col and SALK_084889 seeds were grown in 0.5× MS media (2.2 g of MS basal salts (Sigma), 5 g of Sucrose, 0.5 g of MES, and 1 mL of 1,000× Gamborg vitamins (Sigma) per liter at pH 5.75) at 22 °C under constant light condition. The 14 day old plants were harvested from MS plates and directly preserved in RNALater (Ambion) [2]. For drought treatment, harvested Arabidopsis plants were dehydrated on Whatman 3mm paper (Whatman) at 22 °C and 60% humidity under dim light for 2 h [3] prior to fixation in RNALater. For RNA extraction, root and shoot tissues were dissected in RNALater solution and then total RNA was isolated using the Qiagen RNAeasy kit (Qiagen). Quantitative realtime PCR was performed using Fast SYBR Green Master Mix (Thermo Fisher) and 2^-ΔΔCt^ method was employed to calculate the relative expression levels of *DREB2A and LOX1*. Three replicates were used for each sample and *UBQ11* (AT4G05050) gene was used as the internal control. For drought and heat tolerance determination of SALK_084889, Col and SALK_084889 seeds were grown in soil at 22 °C under constant light condition. For drought test, the 10 day old plants were transferred to pre-dried soil and withhold water for 4 days prior to recovery by watering for 7 days [4]. For heat test, the 7 day old plants were pre-incubated in 37 °C for 1 h and then treated at 49 °C for 1 h prior to recovery in 22 °C for 2 days [5]. Survival rates and phenotypes of Col and SALK_084889 plants were recorded. Student׳s *t*-test was used for statistical analysis.
