# Supplementary material for: Data for characterization of SALK_084889, a T-DNA insertion line of Arabidopsis thaliana
Source: Data Brief. 2017 May 31;13:253–8. doi: 10.1016/j.dib.2017.05.047 (PMC5470433; doi:10.1016/j.dib.2017.05.047)
Supplement: Supplementary file 1 — Supplementary material [file mmc1.docx]

No Conflict of Interest.
